# AI-based diagnosis in mandibulofacial dysostosis with microcephaly using external ear shapes

**DOI:** 10.3389/fped.2023.1171277

**Published:** 2023-08-17

**Authors:** Quentin Hennocq, Thomas Bongibault, Sandrine Marlin, Jeanne Amiel, Tania Attie-Bitach, Geneviève Baujat, Lucile Boutaud, Georges Carpentier, Pierre Corre, Françoise Denoyelle, François Djate Delbrah, Maxime Douillet, Eva Galliani, Wuttichart Kamolvisit, Stanislas Lyonnet, Dan Milea, Véronique Pingault, Thantrira Porntaveetus, Sandrine Touzet-Roumazeille, Marjolaine Willems, Arnaud Picard, Marlène Rio, Nicolas Garcelon, Roman H. Khonsari

**Affiliations:** ^1^ Imagine Institute, INSERM UMR1163, Paris, France; ^2^ Service de Chirurgie Maxillo-Faciale et Chirurgie Plastique, Hôpital Necker—Enfants Malades, Assistance Publique—Hôpitaux de Paris, Centre de Référence des Malformations Rares de la Face et de la Cavité Buccale MAFACE, Filière Maladies Rares TeteCou, Faculté de Médecine, Université de Paris Cité, Paris, France; ^3^ Laboratoire ‘Forme et Croissance du Crâne’, Faculté de Médecine, Hôpital Necker-Enfants Malades, Assistance Publique-Hôpitaux de Paris, Université Paris Cité, Paris, France; ^4^ Service de Médecine Génomique des Maladies Rares, Hôpital Necker—Enfants Malades, Assistance Publique—Hôpitaux de Paris, Faculté de Médecine, Université de Paris Cité, Paris, France; ^5^ CHU Lille, Inserm, Service de Chirurgie Maxillo-Faciale et Stomatologie, U1008-Controlled Drug Delivery Systems and Biomaterial, Université de Lille, Lille, France; ^6^ Department of Oral and Maxillofacial Surgery, INSERM U1229—Regenerative Medicine and Skeleton RMeS, Nantes, France; ^7^ Department of Oral and Maxillofacial Surgery, Nantes University, CHU Nantes, Nantes, France; ^8^ Department of Paediatric Otolaryngology, AP-HP, Hôpital Necker-Enfants Malades, Paris, France; ^9^ Center of Excellence for Medical Genomics, Department of Pediatrics, Faculty of Medicine, Chulalongkorn University, Bangkok, Thailand; ^10^ Center of Excellence in Genomics and Precision Dentistry, Department of Physiology, Faculty of Dentistry, Chulalongkorn University, Bangkok, Thailand; ^11^ Duke-NUS Medical School Singapore, Singapore Eye Research Institute, Singapore National Eye Centre, Singapore, Singapore; ^12^ Département de Génétique Clinique, CHRU de Montpellier, Hôpital Arnaud de Villeneuve, Institute for Neurosciences of Montpellier, INSERM, Univ Montpellier, Montpellier, France

**Keywords:** AI, machine learning, dysmorphology, craniofacial malformation, MFDM

## Abstract

**Introduction:**

Mandibulo-Facial Dysostosis with Microcephaly (MFDM) is a rare disease with a broad spectrum of symptoms, characterized by zygomatic and mandibular hypoplasia, microcephaly, and ear abnormalities. Here, we aimed at describing the external ear phenotype of MFDM patients, and train an Artificial Intelligence (AI)-based model to differentiate MFDM ears from non-syndromic control ears (binary classification), and from ears of the main differential diagnoses of this condition (multi-class classification): Treacher Collins (TC), Nager (NAFD) and CHARGE syndromes.

**Methods:**

The training set contained 1,592 ear photographs, corresponding to 550 patients. We extracted 48 patients completely independent of the training set, with only one photograph per ear per patient. After a CNN-(Convolutional Neural Network) based ear detection, the images were automatically landmarked. Generalized Procrustes Analysis was then performed, along with a dimension reduction using PCA (Principal Component Analysis). The principal components were used as inputs in an eXtreme Gradient Boosting (XGBoost) model, optimized using a 5-fold cross-validation. Finally, the model was tested on an independent validation set.

**Results:**

We trained the model on 1,592 ear photographs, corresponding to 1,296 control ears, 105 MFDM, 33 NAFD, 70 TC and 88 CHARGE syndrome ears. The model detected MFDM with an accuracy of 0.969 [0.838–0.999] (*p* < 0.001) and an AUC (Area Under the Curve) of 0.975 within controls (binary classification). Balanced accuracies were 0.811 [0.648–0.920] (*p* = 0.002) in a first multiclass design (MFDM vs. controls and differential diagnoses) and 0.813 [0.544–0.960] (*p* = 0.003) in a second multiclass design (MFDM vs. differential diagnoses).

**Conclusion:**

This is the first AI-based syndrome detection model in dysmorphology based on the external ear, opening promising clinical applications both for local care and referral, and for expert centers.

## 1. Introduction

Mandibulo-Facial Dysostosis with Microcephaly (MFDM), formerly named Mandibulo-Facial Dysostosis Guion Almeida type (MFDGA) (1, 2), is a rare disease with a broad spectrum of symptoms, characterized by zygomatic (92%) and mandibular (93%) hypoplasia, microcephaly (88%, 64% congenital or 36% postnatal), cognitive impairment (97%–100%), small or dysplastic external ear (97%) and deafness (83%), most often conductive (3). MFDM may also include choanal atresia (30%–33%), cleft palate (43%–47%), facial asymmetry (53%–58%), and extra-facial abnormalities, such as heart malformations (30%–35%), thumb abnormalities (31%), esophageal involvement (atresia/fistulae, 27%–33%), short stature (30%), vertebral abnormalities (28%) and epilepsy (27%) (4). Facial dysostoses are subdivided into two groups: Mandibulo-Facial Dysostoses (MFD) and Acro-Facial Dysostoses (AFD), the latter including limb abnormalities (5). Because there may be associated with spine abnormalities, some authors have listed MFDM as a pre-axial acrofacial dysostosis, Guion Almeida type (AFDGA) (5–7).

Since 2012, the diagnosis of MFDM is established based on clinical features and the screening for a heterozygous pathogenic variant of the *EFTUD2* gene (17q21.31) coding for the nuclear ribonucleoprotein component of 116 KDA U5 protein (8). This variant occurs frequently *de novo* (80%) (4, 9). The main mechanism of disease is haploinsufficiency (10), caused in 18% of cases by a missense substitution, in 38% by a stop-gain *EFTUD2* heterozygous pathogenic variation and in 43% by a splice site variation (4, 11). No genotype-phenotype correlations in patients with *EFTUD2* heterozygous pathogenic variations have been identified (8, 12).

Regarding deformities of the external ear in MFDM, Lines et al. (3, 8) described microtia (grades I-III), abnormalities of the superior helix and antihelix, preauricular tags and auditory canal atresia/stenosis. The posterior-inferior margin of the lobule can have a right-angle (“squared-off”) configuration (3, 8, 13).

The main differential diagnoses of MFDM are other mandibulofacial dysostoses — i.e., Nager type Acro-Facial Dysostosis (NAFD), Postaxial acrofacial dysostosis Miller type, and Treacher Collins (TC) syndromes — and CHARGE syndrome (14, 15). MFDM patients are often misdiagnosed within this spectrum. Distinguishing MFDM ears from CHARGE ears can sometimes be tricky, and *EFTUD2* heterozygous pathogenic variation screening is recommended in patients with unusual forms of CHARGE syndrome (14).

Based on these clinical questions, the three objectives of this study were: (1) objectively determine the phenotype of pinna malformations in MFDM using geometric morphometrics and machine learning techniques vs. controls (design № 1), (2) compare the ears of MFDM patients with ears from the main differential diagnoses, with or without controls (respectively design № 2.1 and № 2.2) and (3) compare phenotypes from the different genotypes causing MFDM (design № 3).

## 2. Material and methods

### 2.1. Training set

We included pictures from the photographic database of the Maxillofacial surgery and Plastic Surgery department and from the Medical genetics department of *Hôpital Necker—Enfants Malades* (Assistance Publique—Hôpitaux de Paris), Paris, France. This database contains 594,000 photographs from 22,000 patients followed in the department since 1981. All photographs were taken by a professional medical photographer using a Nikon D7000 device in standardized positions.

**Figure 1:**
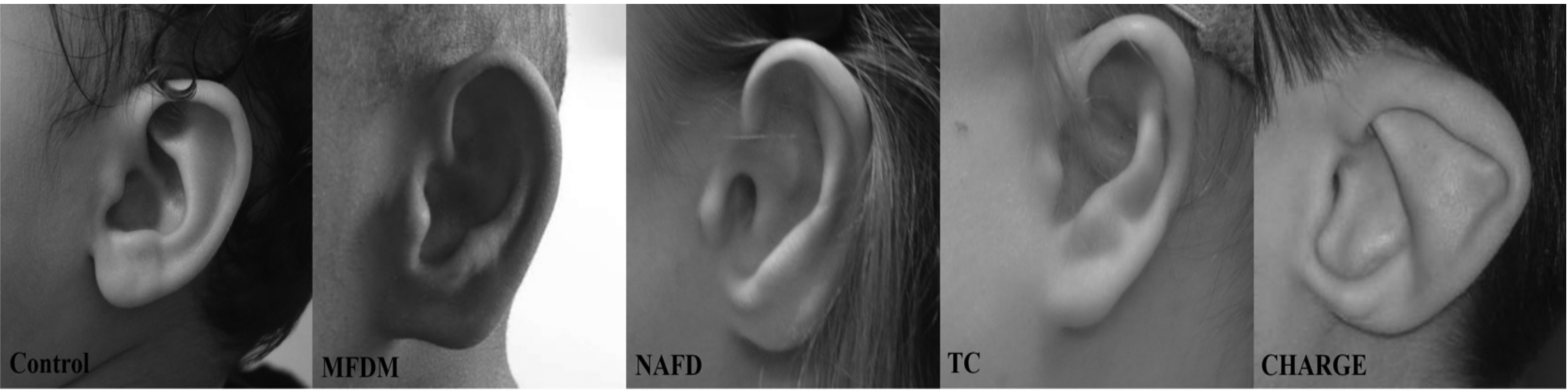
Examples of external ear photographs for each patient group: controls, mandibulo-facial dysostosis with microcephaly (MFDM), Nager type acro-facial dysostosis (NAFD), Treacher Collins (TC), and CHARGE syndromes.

We included retrospectively and prospectively, from 1981 to 2023, all profile pictures of patients diagnosed with MFDM, TC, NAFD and CHARGE syndromes, with a visible pinna (Figure 1). The photographs were not calibrated. All patients had genetic confirmation of their syndrome. We excluded patients with ear reconstruction surgery. Multiple photographs per patient corresponded to different ages. Duplicate photographs were excluded.

Non-syndromic children were selected among patients admitted for wounds, trauma, infection and various skin lesions, without any record of chronic conditions. More precisely, follow-up for any type of chronic disease was considered as an exclusion criterion. The reports were retrieved using Dr Warehouse (16). For each patient, right and left sides were included.

The study was approved by the CESREES (Comité Ethique et Scientifique pour les Recherches, les Etudes et les Evaluations dans le domaine de la Santé, № 4570023bis) and by the CNIL (Commission Nationale Informatique et Libertés, № MLD/MFI/AR221900). Informed and written consents were obtained from the legal representatives of each child, or from the patient himself if he was of age.

### 2.2. Validation set

A fully independent validation set was designed using publicly available data published in the literature. We included patients with MFDM (6, 14, 17), NAFD (18–20), CHARGE syndrome (21–24) and TC syndrome (25, 26); all had genetic confirmation of their syndromes.

We also retrieved ear photographs of these syndromes of interest from the databases of the Maxillofacial surgery and/or Genetics departments of the University Hospitals of Lille (France), Montpellier (France), Nantes (France) and the King Chulalongkorn Memorial Hospital in Bangkok (Thailand). None of the patients in the validation set were present twice, and none were from the training set. For the control group, we selected a group of photographs from our local database, without any redundancy with the training set, using similar inclusion criteria.

We extracted data on age at the time of the photograph and gender. We excluded patients with no information on the contralateral ear to take into account asymmetry or severity.

All photographs in the validation group were manually annotated by two independent raters (QH and MD), blinded for the diagnosis. The ICC (Intraclass Correlation Coefficient) was computed. ICC values greater than 0.9 corresponded to excellent reliability of the manual annotation (27).

### 2.3. Landmarking

We used an available template (28) based on 55 landmarks placed on the outer helix, the antihelix, the lobe, the tragus, the antitragus, the helix, the crus helicis, and the concha. We developed an automatic annotation model trained on 1,592 manually annotated ear photographs following a pipeline including: (1) a Faster R-CNN (Convolution Neural Network) to detect ears on the pixels of lateral face photographs and (2) a patch-AAM (Active Appearance Model), to automatically place landmarks.

The Fast RCNN model (29) was trained on 5,154 ear photographs after data augmentation (1,718 images and their +10° and −10° rotations), with a learning rate of 0.001, a batch size of 4, a gamma of 0.05 and 2,000 iterations. The patch-AAM was trained on 1,221 ear photos, after 50 iterations, with a Lucas-Kanade optimization (30). The Faster R-CNN was developed in Pytorch on Python 3.7 (31). The patch-AAM was developed using the *menpo* library on Python 3.7 (32). These two methods and the choice of hyperparameters have been described in a previous report by our team (33).

Each automatically annotated photograph was checked by the first author (QH) and landmarks were manually re-positioned when necessary, using *landmarker.io* (34).

To ensure a uniform distribution of landmarks along the curves of the ear (outer helix, inner helix, antihelix, concha), anatomical landmarks were transformed into sliding semi-landmarks using the *geomorph* package on R (35). Landmarks corresponding to the antihelix were removed because Hennocq et al. (33) showed that they were not reproducible between two annotators.

Ears were finally annotated based on 41 anatomical landmarks and semi-landmarks, placed automatically and double-checked manually.

### 2.4. Geometric morphometrics

We performed Generalized Procrustes Analysis (GPA) (36) on all landmark clouds using the *geomorph* package on R. Since the data were uncalibrated photographs, ear sizes were not available: shape parameters only were assessed and not centroid sizes.

Procrustes coordinates were processed using Principal Component Analysis (PCA) for dimension reduction (37): 8 principal components (PC) accounting for more than 90% of the global variance were retained.

To take into account associated metadata (age and gender) and the fact that we had included more than one photograph per patient (that is the non-independence of the data), a mixed model was designed for each principal component. The variable to be explained was PC, with age and gender considered as explanatory variables. A random effect on age and individuals was introduced. The equation of the mixed model was:

$$PC_{i,j}∼\;\alpha +\;\mathrm{age}.\beta_{1}+\mathrm{gender}.\beta_{2}+\mathrm{age}.\beta_{1,i}+\varepsilon_{i,j}$$

where $\mathrm{age}.\beta_{1,i}$ corresponded to a random slope for age per individual, and $\varepsilon_{i,j}$ was a random error term. We did not use an interaction term between age and gender as it did not increase the likelihood of the model. Age, gender and ethnicity are significant factors in dysmorphology because they influence the diagnosis, and must therefore be taken into account (38).

### 2.5. Asymmetry and severity of microtia

Accounting for the heterogeneity of external ear anomalies was difficult. We graded microtia in stages I-IV according to the Marx classification (39). Only grade I ears could be annotated, as the main anatomical structures were missing in grades II, III et IV. However, the frequency of ears >grade I had to be considered for each disease group as it was a potential diagnostic feature. Information on the left/right asymmetry was also included as it could have been variable according to syndromes.

The overall severity for each patient was defined as the sum of microtia grades on each ear. Asymmetry was quantified using a mixed scale ranging from 0 to 3, corresponding to the subtraction of the left and right microtia grades. A high score corresponded to high left/right asymmetry. For bilateral grade I ears, we computed an asymmetry index based on fluctuating asymmetry (40, 41), normalized between 0 and 1. A patient with two grade II ears had a symmetry score of 0. A patient with one grade III ear and one grade I ear had a symmetry score of 2. A patient with two grade I ears had an asymmetry score corresponding to his normalized asymmetry index, ranging between 0 and 1.

The severity and asymmetry scores were compared between different groups using mixed linear models to take into account repeated data per patient. The model coefficients for each group were compared to 0 by Student’s t tests. The significance level was set at *p* < 0.05.

### 2.6. Uniform manifold approximation and projection (UMAP) representations

The residuals $\varepsilon_{i,j}$ were represented using UMAP (42), a nonlinear dimension reduction technique for data visualization. Each design was plotted with and without the severity and asymmetry scores. A k (local neighborhood size) value of 15 was used. A cosine metric was introduced to compute distances in high dimensional spaces: the effective minimal distance between embedded points was $10^{-6}$. The three conditions of UMAP, namely uniform distribution, local constancy of the Riemannian metric and local connectivity were verified. UMAP analyses were performed using the package *umap* on R (43).

### 2.7. Machine learning models and metrics

The landmark clouds were superimposed with the previous generalized Procrustes analysis and PCA. With the metadata (age and gender), the residuals $\varepsilon_{i,j}$ were reported for each PC and each ear of the validation group. The inputs to the model were the residuals from the linear models described above.

We used XGBoost (eXtreme Gradient Boosting), a supervised machine learning classifier, for all the analyses (44). We set a number of hyperparameters to improve the performance and effect of the machine learning model: learning rate = 0.3, gamma = 0, maximum tree depth = 6. We separated the dataset into a training set and a testing set, and a 5-fold cross-validation was used to define the ideal number of iterations to avoid overfitting. The model with the lowest logloss-score was chosen for analysis. The chosen model was then used on the independent validation set to test performances, by plotting accuracy, sensitivity, specificity, F1-score, precision and recall, AUC (in a one vs. all design). The ROC (Receiver Operating Characteristics) curves were plotted in R using the *plotROC* package (45).

## 3. Results

### 3.1. Training set

The training set contained 1,592 ear photographs, corresponding to 550 patients; 52% of patients were female and the mean age was 7.2 ± 5.9 years, ranging from 0 to 60.7 years.

We included 1,296 photographs of control ears, corresponding to 471 patients; 53% of controls were female, with a mean age of 7.2 ± 5.4 years.

The MFDM group included 105 photographs from 31 patients, all genetically confirmed (*EFTUD2* heterozygous pathogenic variations); 52% were female and the mean age was 9.2 ± 9.8 years. Regarding ear aplasia, 92% of the ears were normal or grade I, 3% were grade 22, 5% were grade III, and 0% was grade IV.

The NAFD group included 33 pictures from 9 patients, all genetically confirmed (SF3B4), with 56% females, and a mean age of 11.8 ± 8.8 years. All ears were normal or grade I.

We included 70 photographs corresponding to 15 patients in the TC group. The mean age was 5.5 ± 4.2 years and 40% were female. All had genetic confirmation (*TCOF1* or *POLR1D*). Eighty percent of the ears were normal or grade I, 17% grade II, 3% grade III, and 0% grade IV.

The CHARGE group included 88 photos from 24 patients; 42% were female and mean age was 5.1 ±  5.9 years. All were genetically confirmed (*CHD7*). All ears were normal or grade I (Table 1).

**Table 1:** Description of the training set population.

	Total	Controls	MFDM	NAFD	TC	CHARGE
*N* (ears)	1,592	1,296	105	33	70	88
*N* (patients)	550	471	31	9	15	24
Gender						
Female	288/550 (52%)	251/471 (53%)	16/31 (52%)	5/9 (56%)	6/15 (40%)	10/24 (42%)
Age						
Mean ± SD	7.2 ± 5.9	7.2 ± 5.4	9.2 ± 9.8	11.8 ± 8.8	5.5 ± 4.2	5.1 ± 5.9
Median	6.9	7.3	5.0	9.8	5.3	6.5
Min	0.0	0.1	0.0	0.0	0.0	0.0
Max	60.7	60.7	39.6	33.5	17.2	21.6
Identified pathogenic genetic variation		NA	31/31 (100%)	9/9 (100%)	15/15 (100%)	24/24 (100%)
Grade of aplasia						
0–1		1,296/1,296 (100%)	97/105 (92%)	33/33 (100%)	56/70 (80%)	88/88 (100%)
2		0/1,296 (0%)	3/105 (3%)	0/33 (0%)	12/70 (17%)	0/88 (0%)
3		0/1,296 (0%)	5/105 (5%)	0/33 (0%)	2/70 (3%)	0/88 (0%)
4		0/1,296 (0%)	0/105 (0%)	0/33 (0%)	0/70 (0%)	0/88 (0%)

MFDM, mandibulo-facial dysostosis with microcephaly; NAFD, nager type acro-facial dysostosis; TC, treacher collins; CHARGE, coloboma, heart defect, atresia choanae, retarded growth and development, genital hypoplasia, ear anomalies/deafness; SD, standard deviation.

In the MFDM group, 11 out of 31 patients (35%) had a heterozygous pathogenic variation in a splice site of *EFTUD2*. One of these patients had a Lys620Asn variant (1860G > C) which could be considered as a splice site variation and not as missense (35). Nine out of 31 patients (29%) had a frameshift *EFTUD2* heterozygous pathogenic variation, 7/31 (23%) a nonsense variation, and 4/31 (13%) an intragenic deletion. No patient had a missense variation (Supplementary Table S1).

**Figure 2:**
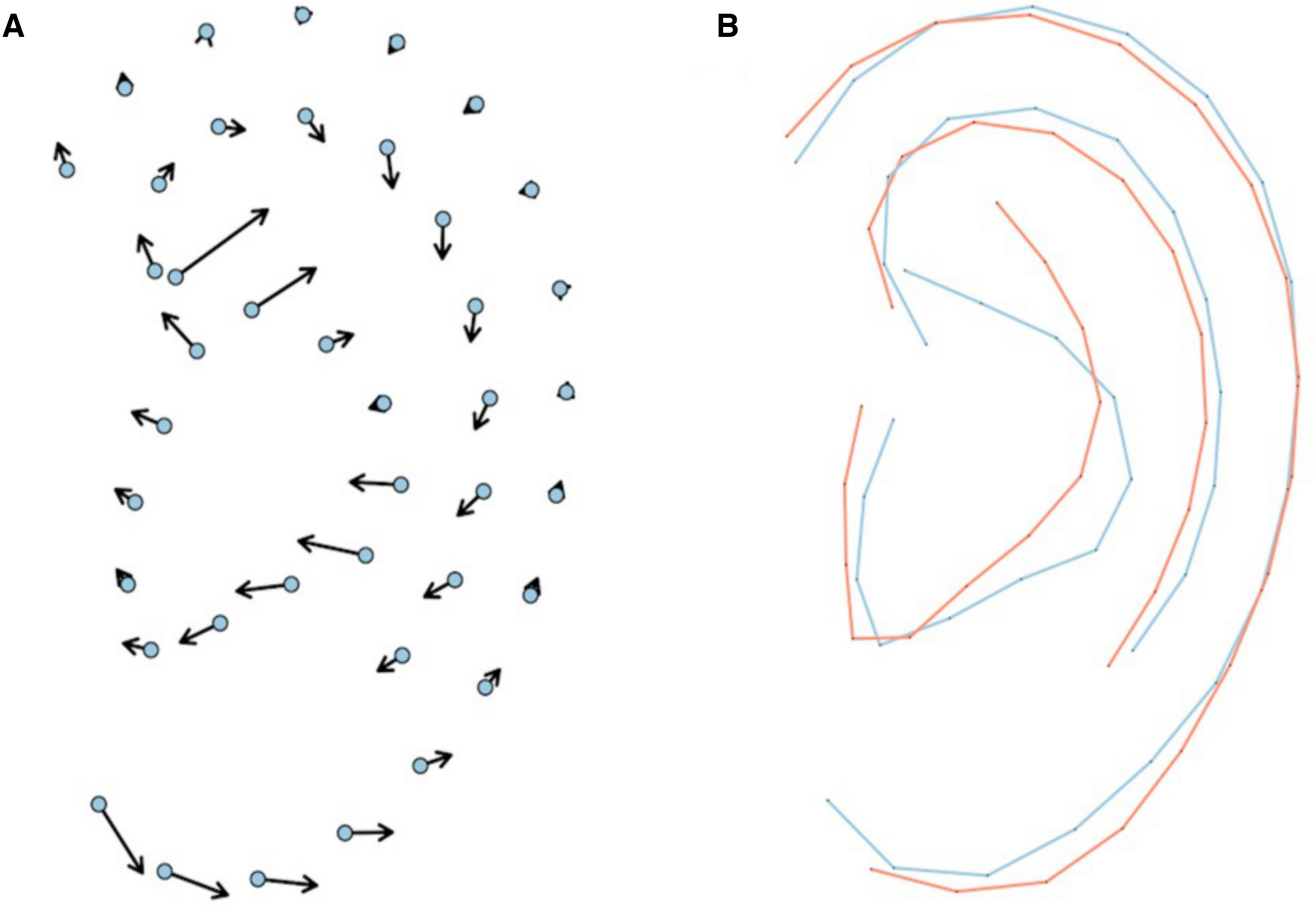
Vectors represent distances between MFDM mean landmarks and the control mean landmarks (**A**). Comparison of average MFDM (red) and control (blue) ear models after Procrustes transformation (**B**).

**Figure 3:**
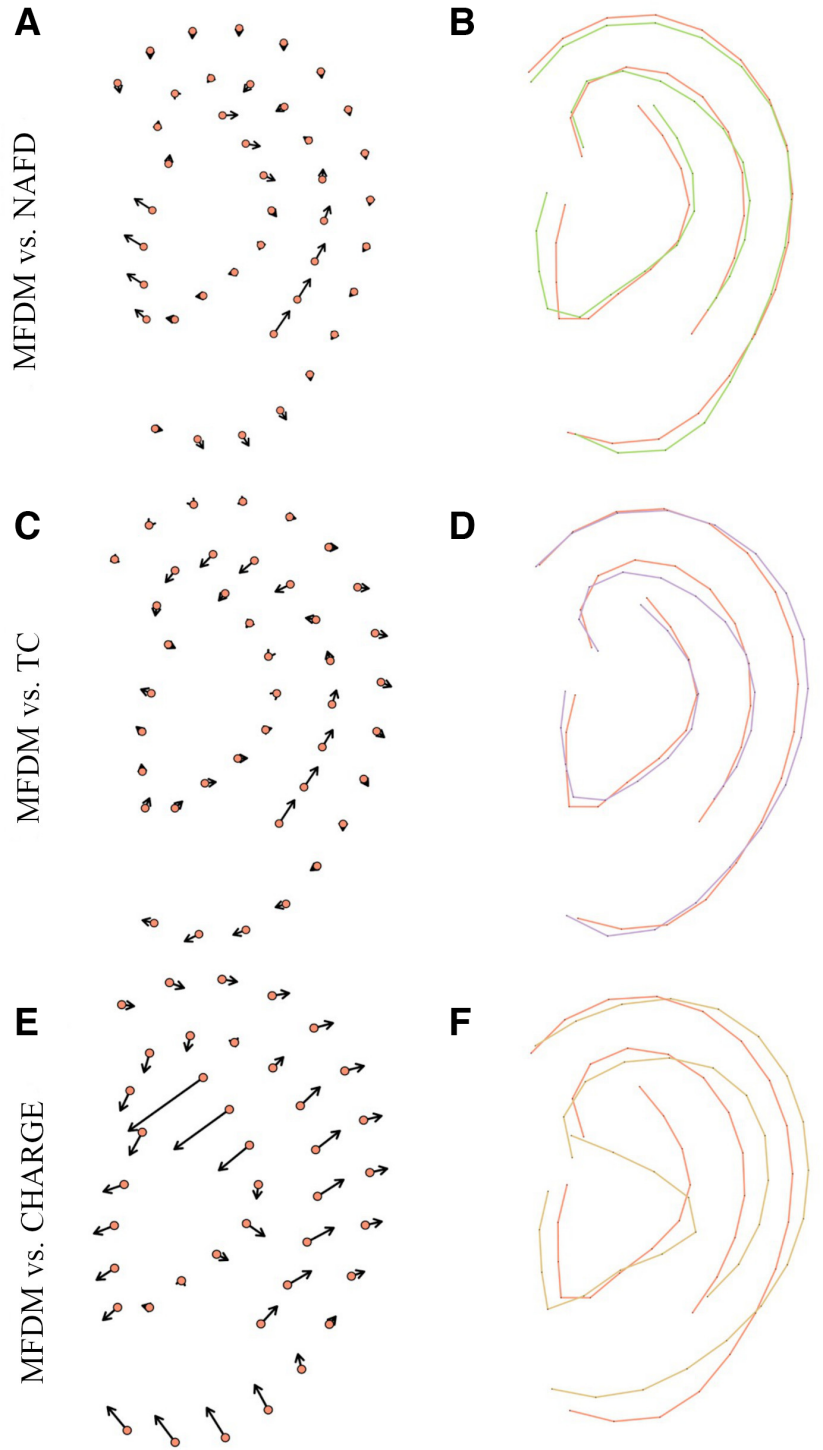
Comparison of average MFDM (red) and the main differential diagnoses: NAFD (green) (**A**, **B**), TC (purple) (**C**, **D**) and CHARGE (yellow) (**E**, **F**), after Procrustes transformation. Vectors (**A**, **C**, **E**) represent distances between MFDM mean landmarks and other groups mean landmarks.

Average models per group were designed after Procrustes transformation, and compared (Figures 2, 3). Ears in the MFDM group had a clockwise rotation and a vertical shift of the concha (Figure 2) when compared to controls. Previously described features—thickened helix, enlarged and square lobe—were also reported.

### 3.2. Validation set

We extracted a total of 48 patients completely independent of the training set, with only one photograph per ear per patient. Severity and asymmetry scores were computed and only one side was then randomly selected. The validation set included 11 MFDM patients (23%), 2 NAFD (4%), 6 TC (13%), 8 CHARGE (17%) and 21 controls (44%) (Supplementary Table S3). We did not have access to the other ear for NAFD patients in the validation set and therefore the asymmetry and severity scores were not obtained.

ICC was 0.991 between the two annotators and the reliability of the annotation was therefore considered as excellent (27).

### 3.3. Severity and asymmetry

Severity and asymmetry scores were compared between groups. In design № 1, TC ears were statistically more severely affected (*p* < 0.001). CHARGE and control groups had lower severity grades (*p* = 0.027 and *p* < 0.001, respectively), compared to MFDM. Control ears were less asymmetric (*p* < 0.001) than MFDM ears. CHARGE ears were less asymmetric than MFDM ears in design № 2.2 (Supplementary Table S2).

### 3.4. UMAP representations

**Figure 4:**
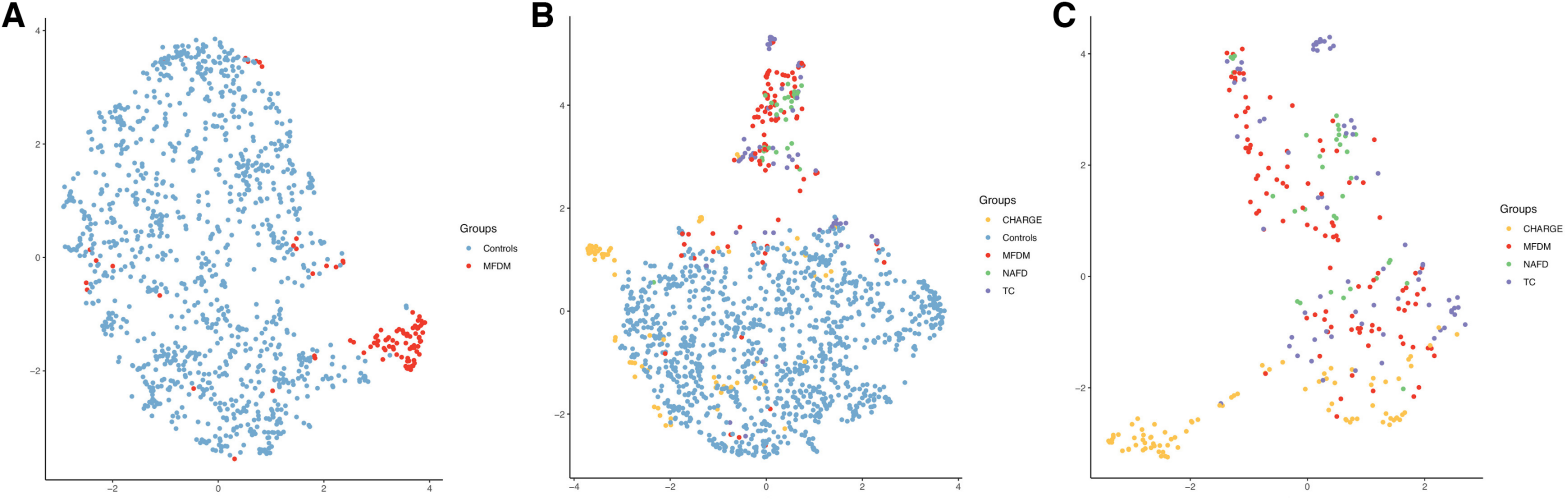
UMAP representations for designs №1 (**A**), № 2.1 (**B**) and № 2.2 (**C**), including severity and asymmetry parameters. Each color corresponds to a patient group. MFDM, Mandibulo-Facial Dysostosis with Microcephaly; NAFD, Nager type Acro-Facial Dysostosis; TC, Treacher Collins; CHARGE, Coloboma, Heart defect, Atresia choanae, Retarded growth and development, Genital hypoplasia, Ear anomalies/deafness.

Patients were clustered using UMAP (Figure 4). MFDM patients were distinct from controls (design № 1, Figure 4A), and CHARGE patients, but not from NAFD and TC patients (designs № 2.1 and № 2.2, Figures 4B,C).

### 3.5. Machine learning models and metrics

#### 3.5.1. Design № 1

**Figure 5:**
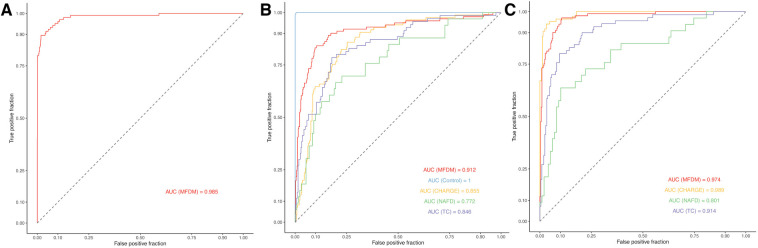
Empirical ROC curves for designs № 1 (**A**), № 2.1 (**B**) and № 2.2 (**C**). MFDM, Mandibulo-Facial Dysostosis with Microcephaly; NAFD, Nager type Acro-Facial Dysostosis; TC, Treacher Collins; CHARGE, Coloboma, Heart defect, Atresia choanae, Retarded growth and development, Genital hypoplasia, Ear anomalies/deafness.

The best performances were obtained without integrating the asymmetry and severity parameters, after 114 iterations. The AUC was 0.985 in the training set (Figure 5A). Patients could be classified into MFDM or control groups in the validation set with a balanced accuracy of 0.969 [0.838–0.999] (*p* < 0.001) and an AUC of 0.975 (Table 2). Only one patient was misclassified (Table 3).

**Table 2:** Classification results on the validation set for design № 1.

Design № 1	
Accuracy	0.969 [0.838–0.999] *p* < 0.001*
Sensitivity (Se)	1.000
Specificity (Sp)	0.909
Balanced Accuracy	0.954
AUC	0.975

Se, Sensitivity; Sp, Specificity.

*Statistically significant test result (*p* ≤ 0.05).

**Table 3:** Confusion matrix on the validation set for design № 1.

		Reference	
		MFDM	Control
Prediction	MFDM	**10**	0
	Control	1	**21**

MFDM, mandibulo-facial dysostosis with microcephaly.

Bolded values denote True Positives (TP).

#### 3.5.2. Design № 2.1

The best performances were obtained by integrating the asymmetry and severity parameters. The classification into MFDM, TC, CHARGE and control groups in the validation set was optimized after 76 iterations. The AUC was 0.912 for MFDM, 1.000 for controls, 0.855 for CHARGE, 0.772 for NAFD and 0.846 for TC in the training set (Figure 5B). On the validation data, the overall balanced accuracy was 0.811 [0.648–0.920] (*p* = 0.002). The balanced accuracy was 0.769 for the classification into MFDM, 0.721 for TC, 0.752 for CHARGE and 0.938 for controls. AUC in the validation set was 0.837 for MFDM, 1.000 for controls, 0.857 for CHARGE and 0.500 for TC (Tables 4, 5).

**Table 4:** Classification results on the validation set for design № 2.1.

Design № 2.1		
Overall (multiclass design)		
Accuracy		0.811 [0.648–0.920] *p* = 0.002*
Binary (one-vs.-all design)		
Sensitivity (Se)	MFDM	0.571
	Control	1.000
	CHARGE	0.571
	TC	0.500
Specificity (Sp)	MFDM	0.967
	Control	0.875
	CHARGE	0.933
	TC	0.943
Balanced Accuracy	MFDM	0.769
	Control	0.938
	CHARGE	0.752
	TC	0.721
AUC	MFDM	0.837
	Control	1.000
	CHARGE	0.857
	TC	0.500

MFDM, mandibulo-facial dysostosis with microcephaly; TC, treacher collins; CHARGE, coloboma, heart defect, atresia choanae, retarded growth and development, genital hypoplasia, ear anomalies/deafness.

*Statistically significant test result (*p* ≤ 0.05).

**Table 5:** Confusion matrix on the validation set for design № 2.1.

		Reference			
		MFDM	Control	CHARGE	TC
Prediction	MFDM	**4**	0	1	0
	Control	0	**21**	2	0
	CHARGE	1	0	**4**	1
	TC	2	0	0	**1**

MFDM, mandibulo-facial dysostosis with microcephaly; TC, treacher collins; CHARGE, coloboma, heart defect, atresia choanae, retarded growth and development, genital hypoplasia, ear anomalies/deafness.

Bolded values denote True Positives (TP).

#### 3.5.3. Design № 2.2

The best performances were obtained by integrating the asymmetry and severity parameters. The classification into MFDM, TC and CHARGE groups in the validation set was optimized after 91 iterations. The AUC was 0.974 for MFDM, 0.889 for CHARGE, 0.801 for NAFD and 0.914 for TC in the training set (Figure 5C). On the validation data, the overall balanced accuracy was 0.813 [0.544–0.960] (*p* = 0.003). With this classifier, the balanced accuracy was 0.944 for the classification into MFDM, 0.873 for CHARGE and 0.500 for TC. AUC in the validation set was 1.000 for MFDM, 0.969 for CHARGE and 0.500 for TC (Tables 6, 7).

**Table 6:** Classification results on the validation set for design № 2.2.

Design № 2.2		
Overall (multiclass design)		
Accuracy		0.813 [0.544–0.960] *p* = 0.003*
Binary (one-vs.-all design)		
Sensitivity (Se)	MFDM	1.000
	CHARGE	0.857
	TC	0.000
Specificity (Sp)	MFDM	0.889
	CHARGE	0.889
	TC	0.929
Balanced accuracy	MFDM	0.944
	CHARGE	0.873
	TC	0.464
AUC	MFDM	1.000
	CHARGE	0.969
	TC	0.500

MFDM, Mandibulo-Facial Dysostosis with Microcephaly; TC, Treacher Collins; CHARGE, Coloboma, Heart defect, Atresia choanae, Retarded growth and development, Genital hypoplasia, Ear anomalies/deafness.

*Statistically significant test result (*p* ≤ 0.05).

**Table 7:** Confusion matrix on the validation set for design № 2.2.

		Reference		
		MFDM	CHARGE	TC
Prediction	MFDM	**7**	0	1
	CHARGE	0	**6**	1
	TC	0	1	**0**

MFDM, mandibulo-facial dysostosis with microcephaly; TC, treacher collins; CHARGE, coloboma, heart defect, atresia choanae, retarded growth and development, genital hypoplasia, ear anomalies/deafness.

Bolded values denote True Positives (TP).

#### 3.5.4. Design № 3

AUC was 0.602 [0.483–0.734] (*p* = 0.370) on the training set. This classification was not statistically significant and was therefore not tested on the validation set. The UMAP representation did not find any clusters based on *EFTUD2* heterozygous pathogenic variation type and site (Supplementary Figure S1).

## 4. Discussion

Applications of machine learning are increasing in healthcare (46–49). The field of dysmorphology has been transformed by the framework for genetic syndrome classification called DeepGestalt (50), produced by the Face2Gene group. Publications comparing human performances to DeepGestalt performances are flourishing (51–54), and some authors state that digital tools provide better results than human experts in terms of diagnosis. We do not believe that Artificial Intelligence (AI) algorithms can fully replace the experience of an expert practitioner, but AI-based tools can considerably increase diagnostic performances, and also contribute to the diffusion of specialized expertise. However, as in all deep learning approaches, DeepGestalt predictions are tricky to explain (50): the phenotypic traits leading to diagnosis cannot be traced. Moreover, only the frontal facial pictures are considered within this framework, that does not take into account the profile pictures and external ears. To our knowledge, we report the first machine learning classifier based on external ear shape. Even though the diagnosis of a given syndrome is never fully based on ear anomalies, this anatomical region is a major source of distinctive phenotypic features in a large array of syndromes (42–44).

Ear phenotype in MFDM has been previously reported. Guion-Almeida et al. described 4 Brazilian children with small ears, a large lobe, and preauricular skin tags in years 2000 (55) and 2006 (1). In 2009 (2), the same team described small and cup-shaped ears with atretic external auditory canal in two other cases. Smigiel et al. (56) reported three MFDM cases with asymmetric microtia, a thickened helix, and protruding ear lobes. Lehalle et al. (17) described abnormalities of the external ear in 100% out of 34 MFDM cases, with minor abnormalities in 29/34 cases (squared, flattened and externally deviated ear lobe), asymmetric ears in 24% of cases and preauricular tags in 33% of cases. Voigt et al. (6), Huang et al. (4), Lines et al. (8) et Yu et al. (57) described similar abnormal pinnae. We could not find any information in the literature on the frequency of grade >I ear involvement in MFDM, or on the asymmetry of microtia.

In TC, Katsanis & Jabs (58) reported absent or small, malformed, sometimes rotated ears. Abdollahi Fakhim et al. (59) compared NAFD and TC without mentioning ears. Bernier et al. (18) described pinnae malformations in NAFD without providing further details. We did not find detailed phenotypic descriptions of the external ear in TC and NAFD in the literature.

In contrast, Davenport et al. (60) described the ear phenotype of CHARGE ears in greater details. CHARGE ears were small, wide and ‘looked as if they were stretched or bent’ (60). The most distinctive feature according to these authors was the triangular shape of the concha and a discontinuity between the antihelix and the antitragus. Davenport et al. (60) also explained that many patients had small or absent lobes, with significant left/right asymmetry.

We thus report new features for MFDM ears: clockwise rotation and vertical shift of the concha (Figure 2). We confirm previously described features such as helix thickening, and enlarged and squared lobes. MDFM ears were also more asymmetric than controls. These overall features were shared with the NAFD and TC groups. Microtia grades were nevertheless higher in TC. CHARGE ears had a specific shape, with a triangular concha, a smaller but wider overall size with a thinner helix and a smaller lobe. In brief, the shape of the pinna can be considered as a relevant feature to differentiate MFDM from CHARGE.

The classification algorithm from design № 1 provides an accuracy of 96.9% for distinguishing MDFM from controls, with only 1 patient misclassified in the validation set. with poorer results when using multi-class classification, which provides an overall balanced accuracy of 81.1% in design № 2.1 (MFDM and its differential diagnoses + controls) and 81.3% in design № 2.2 (MFDM and its differential diagnoses). These results account for the difficulty to diagnose MFDM from NAFD and TC. On the other hand, our results were satisfactory for detecting CHARGE ears, with an AUC reaching 85.7% in design № 2.1, and 96.9% in design № 2.2. We could not detect any genotype-phenotype correlations (design № 3).

**Figure 6:**
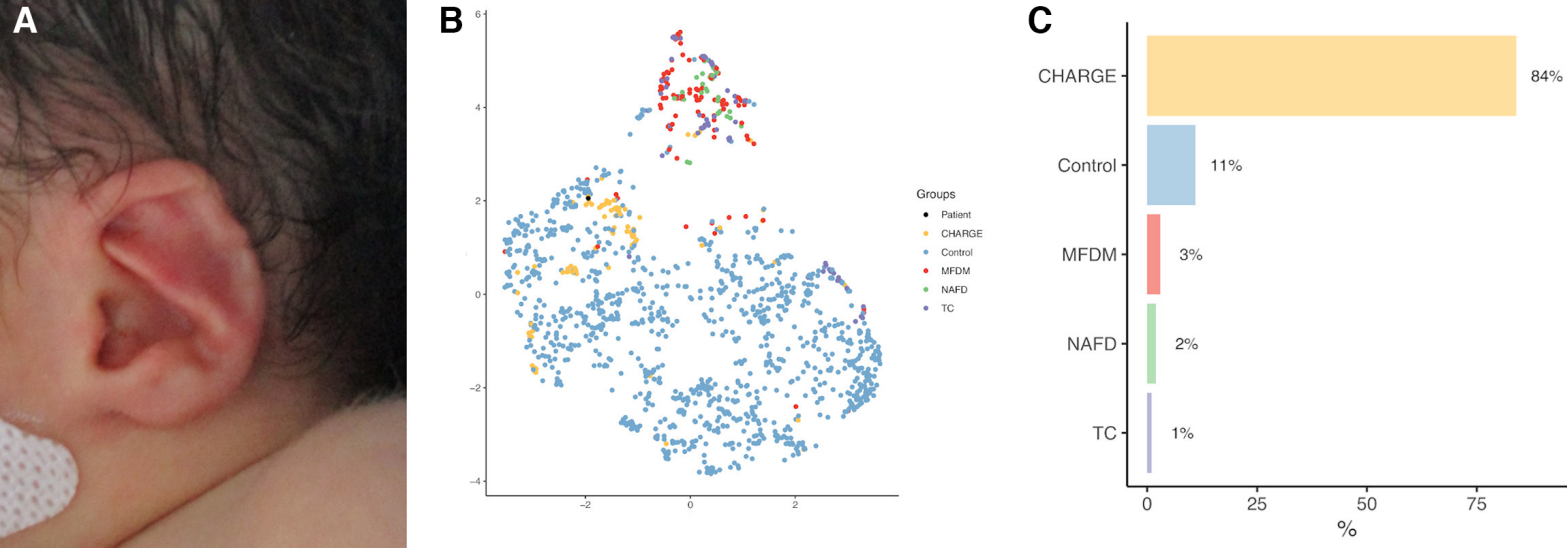
Case study of automatic ear-based CHARGE syndrome diagnosis (**A**). (**B**) UMAP clustering of design № 2.1; black dot: patient. (**C**) probability histogram with a XGboost classifier.

The clinical use of automatic ear-based diagnosis can be highlighted based on a preliminary case study. A non-premature female child aged 9 days was admitted in fetal pathology with bilateral choanal atresia, inner ear malformations, agenesis of the acoustic-facial bundle and cerebellopontine hypoplasia. She had died within a few days after birth. CHARGE syndrome was confirmed post-mortem by a heterozygous *de novo* pathogenic variation in the *CHD7* gene (c. 4,353 + 1G > A). The patient also carried a heterozygous *de novo* variation of unknown significance in the *EFTUD2* gene (c. 1954G > A, p.Asp652Asn). Our ear-based model on the ears of this patient (with a XGBoost classifier) proposed: CHARGE syndrome 84%, control patient 11%, MFDM 3%, NAFD 2% or TC 1% (Figure 6), supporting the diagnosis of CHARGE syndrome, and showing little tendency towards MFDM ear. As systematic *EFTUD2* heterozygous pathogenic variation screening being currently recommended in unusual CHARGE cases [9], our model, with further clinical validation, could be used as a clinical support for directing genetic investigations.

Here we report the first attempt of automatic ear-based diagnosis in craniofacial dysmorphology. The algorithms we propose have been tested on independent and international validation sets involving rare disease centers in Europe and Asia. Validation data was nevertheless limited for NAFD, highlighting the need for data sharing when designing machine learning-based clinical tools. AI-based automatic facial diagnostic algorithms, including profile and ear analysis, are powerful approaches in supporting practitioners in diagnostic processes.

## Data Availability

The datasets presented in this study can be found in online repositories. The names of the repository/repositories and accession number(s) can be found in the article/**Supplementary Material**.

## Supplementary material

Supplementary Figure S1 UMAP representations for design №3. (**A**). Type of variation. (**B**). Site of variation on EFTUD2: first half (“beginning”) or second half (“end”).

Supplementary Table S1

EFTUD2 heterozygous pathogenic variations in patients with MFDM MFDM, Mandibulo-Facial Dysostosis with Microcephaly.

Supplementary Table S2

Comparisons of severity and asymmetry scores by study design. MFDM, Mandibulo-Facial Dysostosis with Microcephaly; NAFD, Nager type Acro-Facial Dysostosis; TC, Treacher Collins; CHARGE, Coloboma, Heart defect, Atresia choanae, Retarded growth and development, Genital hypoplasia, Ear anomalies/deafness.

Supplementary Table S3

Description of the validation set population. MFDM, Mandibulo-Facial Dysostosis Guion Almeida type; NAFD, Nager type Acro-Facial Dysostosis; TC, Treacher Collins; CHARGE, Coloboma, Heart defect, Atresia choanae, Retarded growth and development, Genital hypoplasia, Ear anomalies/deafness; SD, Standard Deviation.

## References

[1] Guion-Almeida ML Zechi-Ceide RM Vendramini S Ju Nior AT. A new syndrome with growth and mental retardation, mandibulofacial dysostosis, microcephaly, and cleft palate. Clin Dysmorphol. (2006) 15(3):171–4. 10.1097/01.mcd.0000220603.09661.7e 16760738

[2] Guion-Almeida ML Vendramini-Pittoli S Passos-Bueno MRS Zechi-Ceide RM. Mandibulofacial syndrome with growth and mental retardation, microcephaly, ear anomalies with skin tags, and cleft palate in a mother and her son: autosomal dominant or X-linked syndrome? Am J Med Genet A. (2009) 149A(12):2762–4. 10.1002/ajmg.a.32816 19921636

[3] Lines M Hartley T MacDonald SK Boycott KM. Mandibulofacial dysostosis with microcephaly. In: Adam MP Ardinger HH Pagon RA Wallace SE Bean LJ Gripp KW, editors. Genereviews®. Seattle (WA): University of Washington (1993). (Cited 2022 May 25). Available at: http://www.ncbi.nlm.nih.gov/books/NBK214367/24999515

[4] Huang L Vanstone MR Hartley T Osmond M Barrowman N Allanson J Mandibulofacial dysostosis with microcephaly: mutation and database update. Hum Mutat. (2016) 37(2):148–54. 10.1002/humu.22924 26507355 PMC5512564

[5] Wieczorek D. Human facial dysostoses. Clin Genet. (2013) 83(6):499–510. 10.1111/cge.12123 23565775

[6] Voigt C Mégarbané A Neveling K Czeschik JC Albrecht B Callewaert B Oto-facial syndrome and esophageal atresia, intellectual disability and zygomatic anomalies—expanding the phenotypes associated with EFTUD2 mutations. Orphanet J Rare Dis. (2013) 8:110. 10.1186/1750-1172-8-110 23879989 PMC3727992

[7] Bukowska-Olech E Materna-Kiryluk A Walczak-Sztulpa J Popiel D Badura-Stronka M Koczyk G Targeted next-generation sequencing in the diagnosis of facial dysostoses. Front Genet. (2020) 11:580477. 10.3389/fgene.2020.580477 33262786 PMC7686794

[8] Lines MA Huang L Schwartzentruber J Douglas SL Lynch DC Beaulieu C Haploinsufficiency of a spliceosomal GTPase encoded by EFTUD2 causes mandibulofacial dysostosis with microcephaly. Am J Hum Genet. (2012) 90(2):369–77. 10.1016/j.ajhg.2011.12.023 22305528 PMC3276671

[9] RESERVES IUTD. Orphanet: mandibulofacial dysostosis guion almeida type (Cited 2022 May 13). Available at: https://www.orpha.net/consor/cgi-bin/Disease_Search.php?lng=FR&data_id=11150&Disease_Disease_Search_diseaseType=ORPHA&Disease_Disease_Search_diseaseGroup=79113&Disease(s)/group%20of%20diseases=Mandibulofacial-dysostosis–Guion-Almeida-type&title=Mandibulofacial-dysostosis–Guion-Almeida-type&search=Disease_Search_Simple

[10] Ryu JH Kim HY Ko JM Kim MJ Seong MW Choi BY Clinical and molecular delineation of mandibulofacial dysostosis with microcephaly in six Korean patients: when to consider EFTUD2 analysis? Eur J Med Genet. (2022) 65(5):104478. 10.1016/j.ejmg.2022.104478 35395430

[11] Kim SY Lee DH Han JH Choi BY. Novel splice site pathogenic variant of EFTUD2 is associated with mandibulofacial dysostosis with microcephaly and extracranial symptoms in Korea. Diagnostics (Basel). (2020) 10(5):296. 10.3390/diagnostics10050296 32408545 PMC7277841

[12] Lines M Hartley T MacDonald SK Boycott KM Mandibulofacial dysostosis with microcephaly. In: Adam MP Mirzaa GM Pagon RA Wallace SE Bean LJ Gripp KW, editors. Genereviews®. Seattle (WA): University of Washington (1993). (cited 2023 May 13). Available at: http://www.ncbi.nlm.nih.gov/books/NBK214367/24999515

[13] Silva JB Soares D Leão M Santos H. Mandibulofacial dysostosis with microcephaly: a syndrome to remember. BMJ Case Rep. (2019) 12(8):e229831. 10.1136/bcr-2019-229831 31413053 PMC6700533

[14] Luquetti DV Hing AV Rieder MJ Nickerson DA Turner EH Smith J “Mandibulofacial dysostosis with microcephaly” caused by EFTUD2 mutations: expanding the phenotype. Am J Med Genet A. (2013) 161A(1):108–13. 10.1002/ajmg.a.35696 23239648 PMC3535578

[15] Lacour JC McBride L St Hilaire H Mundinger GS Moses M Koon J Novel *de novo* EFTUD2 mutations in 2 cases with MFDM, initially suspected to have alternative craniofacial diagnoses. Cleft Palate Craniofac J. (2019) 56(5):674–8. 10.1177/1055665618806379 30343593

[16] Garcelon N Neuraz A Salomon R Faour H Benoit V Delapalme A A clinician friendly data warehouse oriented toward narrative reports: dr. Warehouse. J Biomed Inform. (2018) 80:52–63. 10.1016/j.jbi.2018.02.019 29501921

[17] Lehalle D Gordon CT Oufadem M Goudefroye G Boutaud L Alessandri JL Delineation of EFTUD2 haploinsufficiency-related phenotypes through a series of 36 patients. Hum Mutat. (2014) 35(4):478–85. 10.1002/humu.22517 24470203

[18] Bernier FP Caluseriu O Ng S Schwartzentruber J Buckingham KJ Innes AM Haploinsufficiency of SF3B4, a component of the pre-mRNA spliceosomal complex, causes Nager syndrome. Am J Hum Genet. (2012) 90(5):925–33. 10.1016/j.ajhg.2012.04.004 22541558 PMC3376638

[19] Gorlin RJ Cohen MM Jr Hennekam RCM. Syndromes of the head and neck. Oxford, UK: Oxford University Press (2001). 1332 p.

[20] Zhao J Yang L. Broad-spectrum next-generation sequencing-based diagnosis of a case of Nager syndrome. J Clin Lab Anal. (2020) 34(9):e23426. 10.1002/jcla.23426 32537850 PMC7521291

[21] Chang JH Park DH Shin JP Kim IT. Two cases of CHARGE syndrome with multiple congenital anomalies. Int Ophthalmol. (2014) 34(3):623–7. 10.1007/s10792-013-9817-4 23807150

[22] Husu E Hove HD Farholt S Bille M Tranebjærg L Vogel I Phenotype in 18 Danish subjects with genetically verified CHARGE syndrome. Clin Genet. (2013) 83(2):125–34. 10.1111/j.1399-0004.2012.01884.x 22462537

[23] Blake KD Prasad C. CHARGE Syndrome. Orphanet J Rare Dis. (2006) 1:34. 10.1186/1750-1172-1-34 16959034 PMC1586184

[24] Lalani SR Safiullah AM Fernbach SD Harutyunyan KG Thaller C Peterson LE Spectrum of CHD7 mutations in 110 individuals with CHARGE syndrome and genotype-phenotype correlation. Am J Hum Genet. (2006) 78(2):303–14. 10.1086/500273 16400610 PMC1380237

[25] Marszałek-Kruk BA Wójcicki P Dowgierd K Śmigiel R. Treacher collins syndrome: genetics, clinical features and management. Genes (Basel). (2021) 12(9):1392. 10.3390/genes12091392 34573374 PMC8470852

[26] Liu J Dong J Li P Duan W. *De novo* TCOF1 mutation in treacher collins syndrome. Int J Pediatr Otorhinolaryngol. (2021) 147:110765. 10.1016/j.ijporl.2021.110765 34058530

[27] Bartko JJ. The intraclass correlation coefficient as a measure of reliability. Psychol Rep. (1966) 19(1):3–11. 10.2466/pr0.1966.19.1.3 5942109

[28] Zhou Y Zaferiou S. Deformable models of ears in-the-wild for alignment and recognition. In: 2017 12th IEEE international conference on automatic face gesture recognition (FG 2017), London, UK. (2017). p. 626–33.

[29] Ren S He K Girshick R Sun J. Faster R-CNN: towards real-time object detection with region proposal networks. IEEE Trans Pattern Anal Mach Intell. (2017) 39(6):1137–49. 10.1109/TPAMI.2016.2577031 27295650

[30] Lucas B Kanade T. An iterative image registration technique with an application to stereo vision (IJCAI). In: IJCAI'81: 7th international joint conference on artificial intelligence, Vol. 2. Pittsburgh, Pennsylvania: ACM Digital Library (1981). p. 674–9.

[31] Paszke A, Gross S, Massa F, Lerer A, Bradbury J, Chanan G http://arxiv.org/abs/1912.01703.

[32] Alabort-i-Medina J Antonakos E Booth J Snape P Zafeiriou S. Menpo: a comprehensive platform for parametric image alignment and visual deformable models. In: Proceedings of the 22nd ACM international conference on multimedia. Orlando Florida USA: ACM Digital Library (2014) p. 679–82. (Cited 2022 Feb 24). Available at: https://dl.acm.org/doi/10.1145/2647868.2654890

[33] Hennocq Q Bongibault T Bizière M Delassus O Douillet M Cormier-Daire V An automatic facial landmarking for children with rare diseases. Am J Med Genet A. (2023) 191(5):1210–21. 10.1002/ajmg.a.63126 36714960

[34] Landmarker.io. The menpo project. (Cited 2022 Mar 20) Available at: https://www.menpo.org/landmarkerio/

[35] Baken EK Collyer ML Kaliontzopoulou A Adams DC. Geomorph v4.0 and gmShiny: enhanced analytics and a new graphical interface for a comprehensive morphometric experience. Methods Ecol Evol. (2021) 12(12):2355–63. 10.1111/2041-210X.13723

[36] Rohlf FJ Slice D. Extensions of the procrustes method for the optimal superimposition of landmarks. Syst Zool. (1990) 39(1):40–59. 10.2307/2992207

[37] Pearson K. LIII On lines and planes of closest fit to systems of points in space. Lond Edinb,Dublin Philos Mag J Sci. (1901) 2(11):559–72. 10.1080/14786440109462720

[38] Burchard EG Ziv E Coyle N Gomez SL Tang H Karter AJ The importance of race and ethnic background in biomedical research and clinical practice. N Engl J Med. (2003) 348(12):1170–5. 10.1056/NEJMsb025007 12646676

[39] Marx H. Die missblindungen des ohreds. Handb Spez Pathol Anat Histol. (1926) 12:620–5. ISBN: 978-3-642-66019-1.

[40] Klingenberg CP Barluenga M Meyer A. Shape analysis of symmetric structures: quantifying variation among individuals and asymmetry. Evolution. (2002) 56(10):1909–20. 10.1111/j.0014-3820.2002.tb00117.x 12449478

[41] Palmer AR. Fluctuating asymmetry analyses: a primer. In: Markow TA, editor. Developmental instability: its origins and evolutionary implications: proceedings of the international conference on developmental instability: its origins and evolutionary implications, tempe, Arizona, 14–15 June 1993. Dordrecht: Springer Netherlands (1994). p. 335–64. (Contemporary Issues in Genetics and Evolution). Available at: doi: 10.1007/978-94-011-0830-0_26 (Cited 2022 Aug 30).

[42] McInnes L Healy J Melville J. UMAP: uniform manifold approximation and projection for dimension reduction. arXiv. (2020). (Cited 2022 Aug 30). Available at: http://arxiv.org/abs/1802.03426

[43] R Core Team. European environment agency. (2020). (Cited 2023 Jan 18). Available at: https://www.eea.europa.eu/data-and-maps/indicators/oxygen-consuming-substances-in-rivers/r-development-core-team-2006

[44] Chen T Guestrin C. XGBoost: a scalable tree boosting system. In: Proceedings of the 22nd ACM SIGKDD international conference on knowledge discovery and data mining. New York, NY, USA: Association for Computing Machinery (2016). p. 785–94. (KDD ‘16). Available at: https://dl.acm.org/doi/10.1145/2939672.2939785 (Cited 2023 Jul 4).

[45] Sachs MC. plotROC: a tool for plotting ROC curves. J Stat Softw. (2017) 79:2. 10.18637/jss.v079.c02 30686944 PMC6347406

[46] Rajkomar A Dean J Kohane I. Machine learning in medicine. N Engl J Med. (2019) 380(14):1347–58. 10.1056/NEJMra1814259 30943338

[47] Choy G Khalilzadeh O Michalski M Do S Samir AE Pianykh OS Current applications and future impact of machine learning in radiology. Radiol. (2018) 288(2):318–28. 10.1148/radiol.2018171820 PMC6542626 29944078

[48] Novoa RA Gevaert O Ko JM. Marking the path toward artificial intelligence-based image classification in dermatology. JAMA Dermatol. (2019) 155(10):1105–6. 10.1001/jamadermatol.2019.1633 31411643

[49] Loftus TJ Tighe PJ Filiberto AC Efron PA Brakenridge SC Mohr AM Artificial intelligence and surgical decision-making. JAMA Surg. (2020) 155(2):148–58. 10.1001/jamasurg.2019.4917 31825465 PMC7286802

[50] Gurovich Y Hanani Y Bar O Nadav G Fleischer N Gelbman D Identifying facial phenotypes of genetic disorders using deep learning. Nat Med. (2019) 25(1):60–4. 10.1038/s41591-018-0279-0 30617323

[51] Zhang Q Ding Y Feng B Tang Y Chen Y Wang Y Molecular and phenotypic expansion of alström syndrome in Chinese patients. Front Genet. (2022) 13:808919. 10.3389/fgene.2022.808919 35211159 PMC8861322

[52] Javitt MJ Vanner EA Grajewski AL Chang TC. Evaluation of a computer-based facial dysmorphology analysis algorithm (Face2Gene) using standardized textbook photos. Eye. (2022) 36(4):859–61. 10.1038/s41433-021-01563-5 33931761 PMC8086228

[53] Latorre-Pellicer A Ascaso Á Trujillano L Gil-Salvador M Arnedo M Lucia-Campos C Evaluating Face2Gene as a tool to identify cornelia de lange syndrome by facial phenotypes. Int J Mol Sci. (2020) 21(3):E1042. 10.3390/ijms21031042 PMC7038094 32033219

[54] Mishima H Suzuki H Doi M Miyazaki M Watanabe S Matsumoto T Evaluation of Face2Gene using facial images of patients with congenital dysmorphic syndromes recruited in Japan. J Hum Genet. (2019) 64(8):789–94. 10.1038/s10038-019-0619-z 31138847

[55] Guion-Almeida ML Kokitsu-Nakata NM Richieri-Costa A. Clinical variability in cerebro-oculo-nasal syndrome: report on two additional cases. Clin Dysmorphol. (2000) 9(4):253–7. 10.1097/00019605-200009040-00004 11045580

[56] Smigiel R Bezniakow N Jakubiak A Błoch M Patkowski D Obersztyn E Phenotype analysis of Polish patients with mandibulofacial dysostosis type guion-almeida associated with esophageal atresia and choanal atresia caused by EFTUD2 gene mutations. J Appl Genet. (2015) 56(2):199–204. 10.1007/s13353-014-0255-4 25387991

[57] Yu KPT Luk HM Gordon CT Fung G Oufadem M Garcia-Barcelo MM Mandibulofacial dysostosis guion-almeida type caused by novel EFTUD2 splice site variants in two Asian children. Clin Dysmorphol. (2018) 27(2):31–5. 10.1097/MCD.0000000000000214 29381487

[58] Katsanis SH Jabs EW. Treacher collins syndrome. In: Adam MP Everman DB Mirzaa GM Pagon RA Wallace SE Bean LJ, editors. Genereviews®. Seattle (WA): University of Washington (1993). (Cited 2022 Sep 8). Available at: http://www.ncbi.nlm.nih.gov/books/NBK1532/20301704

[59] Fakhim S A Shahidi N Mousaviagdas M. A case report: nager acrofacial dysostosis. Iran J Otorhinolaryngol. (2012) 24(66):45–50. PMCID: . 24303385 PMC3846201

[60] Davenport SL Hefner MA Thelin JW. CHARGE Syndrome. Part I. External ear anomalies. Int J Pediatr Otorhinolaryngol. (1986) 12(2):137–43. 10.1016/S0165-5876(86)80071-4 3570680

