# Supplementary figures and images for: AI-based diagnosis in mandibulofacial dysostosis with microcephaly using external ear shapes
Source: Front Pediatr. 2023 Aug 17;11:1171277. doi: 10.3389/fped.2023.1171277 (PMC10469912; doi:10.3389/fped.2023.1171277)

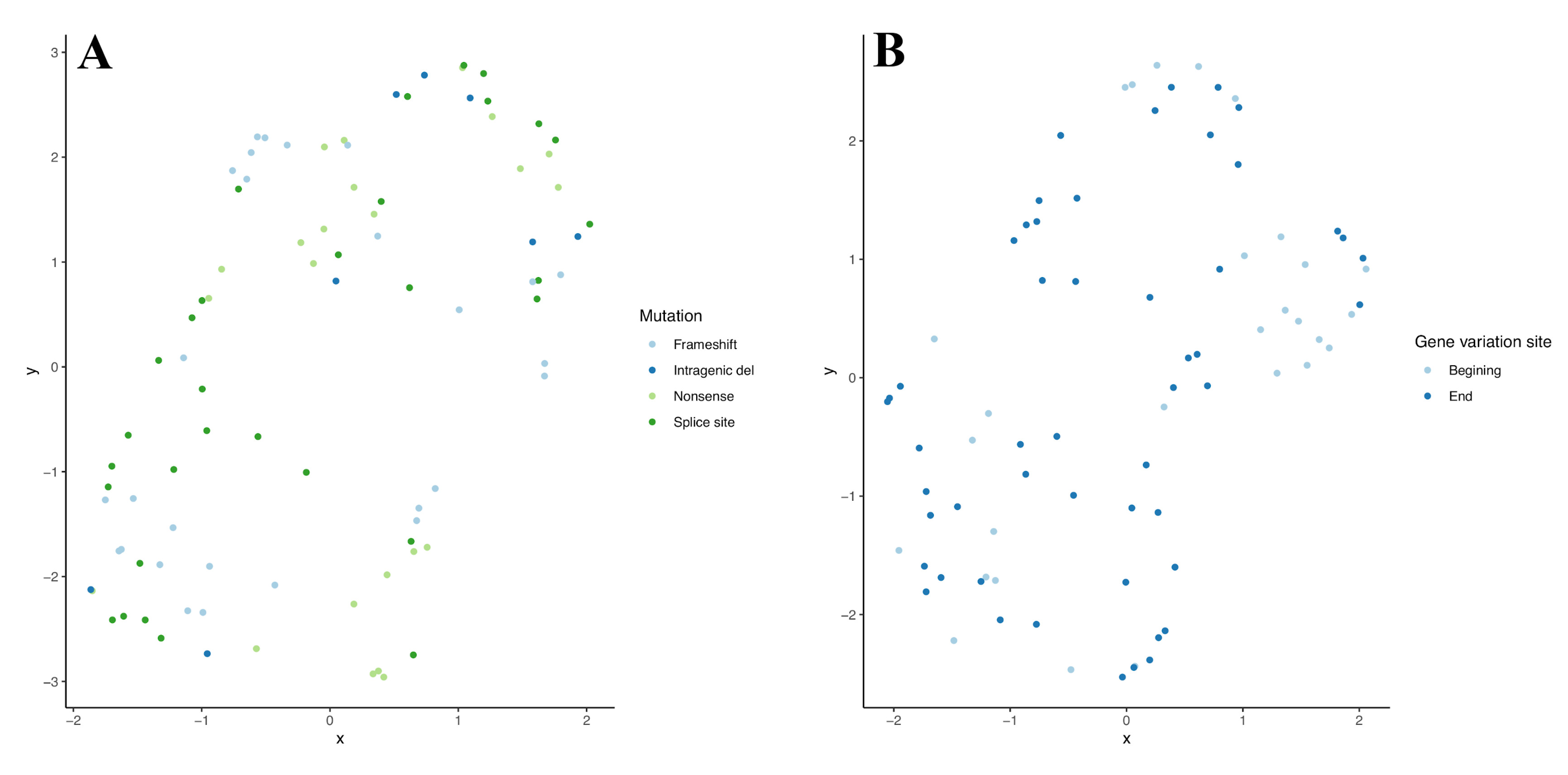

Supplement: Supplementary Figure S1 — UMAP representations for design №3. (A). Type of variation. (B). Site of variation on EFTUD2: first half (“beginning”) or second half (“end”). [file Image1.jpeg]

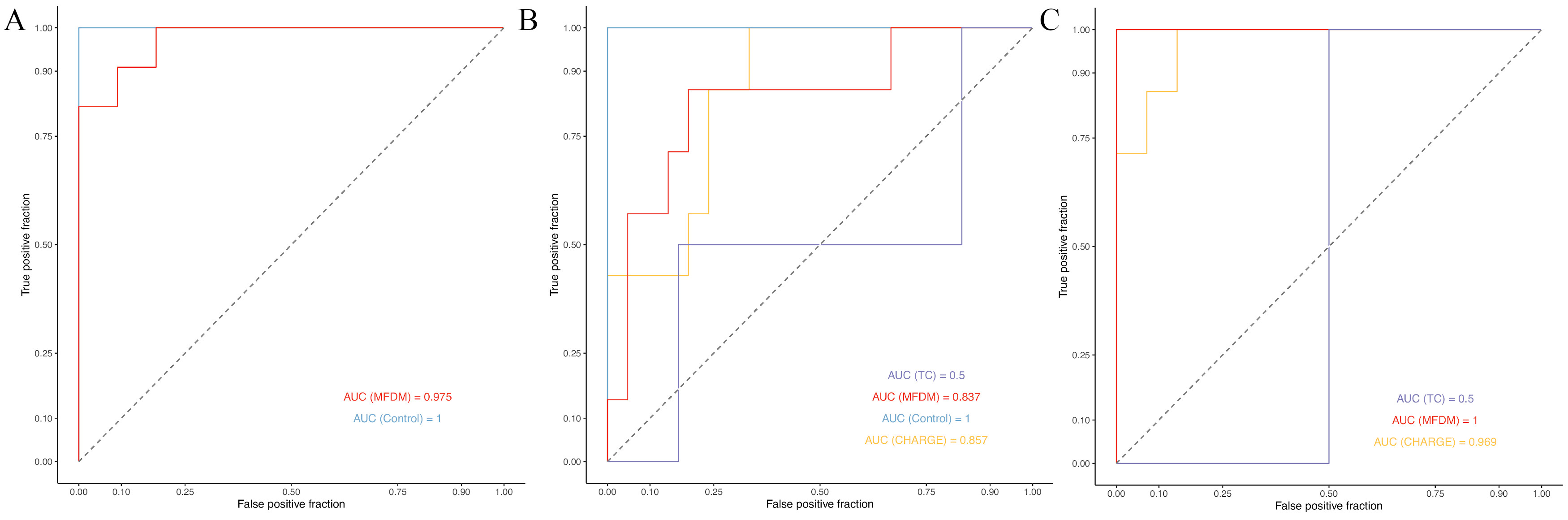

Supplement: Supplementary Figure S2 [file Image2.jpeg]
