# Supplementary material for: AI-based diagnosis in mandibulofacial dysostosis with microcephaly using external ear shapes
Source: Front Pediatr. 2023 Aug 17;11:1171277. doi: 10.3389/fped.2023.1171277 (PMC10469912; doi:10.3389/fped.2023.1171277)
Supplement: Supplementary file 3 [file Table1.docx]

$${PC}_{i,j} \sim\alpha+ age. \beta_{1}+gender.\beta_{2}+ age.\beta_{1,i}+ \varepsilon_{i,j}$$

$age.\beta_{1,i}$ $\varepsilon_{i,j}$ $\varepsilon_{i,j}$ ${10}^{-6}$ $\varepsilon_{i,j}$**Table S1. *EFTUD2* heterozygous pathogenic variations in patients with MFDM.** MFDM = Mandibulo-Facial Dysostosis with Microcephaly

| Variation type | Codon | Variant |
| --- | --- | --- |
| Splice site |  | 702+5G>C |
| Splice site |  | 1149+5G>C |
| Splice site |  | 2133-2A>G |
| Splice site |  | 2562-2del_2562-1del |
| Splice site |  | 351-1G>C |
| Splice site |  | 870-1G>A |
| Splice site |  | 2823+1del |
| Splice site |  | 2562-2del |
| Splice site |  | 995-1G>A |
| Splice site |  | 2133-1G>T |
| Splice site* | Lys620Asn | 1860G>C |
| Frameshift | Thr619Pro*fs**10 | 1854del |
| Frameshift | Gly345Val*fs**41 | 1034del |
| Frameshift | Glu87Ala*fs**8 | 258_259delTC |
| Frameshift | Glu87Ala*fs**8 | 258_259delTC |
| Frameshift | Val593Ala*fs**12 | 1775_1779del |
| Frameshift | Gly806Ala*fs*X22 | 2417del |
| Frameshift | Leu134Pro*fs**18 | c400dup |
| Frameshift | Val81His*fs**16 | 237_240dup |
| Frameshift | Ile766Ser*fs**18 | 2296del |
| Nonsense | Gln152* | 454C>T |
| Nonsense | Glu249X | 745G>T |
| Nonsense | Tyr66* | 198C>G |
| Nonsense | Tyr614* | 1838_1841dup |
| Nonsense | Arg354* | 1060C>T |
| Nonsense | Tyr198* | 594T>A |
| Nonsense | Tyr198* | 594T>A |
| Intragenic deletion | | Deletion of exons 21 to 28 |
| Intragenic deletion | | Deletion of exons 8 to 12 |
| Intragenic deletion | | Deletion of exons 10 to 13 |
| Intragenic deletion | | Deletion of exons 8 to 12 |

**Table S2. Comparisons of severity and asymmetry scores by study design.** MFDM = Mandibulo-Facial Dysostosis with Microcephaly; NAFD = Nager type Acro-Facial Dysostosis; TC = Treacher Collins; CHARGE = Coloboma, Heart defect, Atresia choanae, Retarded growth and development, Genital hypoplasia, Ear anomalies/deafness.

|  |  | Design №1 | Design №2.1 | Design №2.2 |
| --- | --- | --- | --- | --- |
| Severity | MFDM (ref) |  |  |  |
|  | Controls | < 0.001 (-) | < 0.001 (-) |  |
|  | NAFD |  | 0.111 | 0.791 |
|  | TC |  | < 0.001 (+) | < 0.001 (+) |
|  | CHARGE |  | 0.027 (-) | 0.051 |
| Asymmetry | MFDM (ref) |  |  |  |
|  | Controls | < 0.001 (-) | < 0.001 (-) |  |
|  | NAFD |  | 0.518 | 0.897 |
|  | TC |  | 0.537 | 0.147 |
|  | CHARGE |  | 0.172 | 0.011 (-) |

**Table S3. Description of the validation set population.** MFDM = Mandibulo-Facial Dysostosis Guion Almeida type; NAFD = Nager type Acro-Facial Dysostosis; TC = Treacher Collins; CHARGE = Coloboma, Heart defect, Atresia choanae, Retarded growth and development, Genital hypoplasia, Ear anomalies/deafness; SD = Standard Deviation.

|  |  | Total | Controls | MFDM | NAFD | TC | CHARGE |
| --- | --- | --- | --- | --- | --- | --- | --- |
| N (patients) | | 48 | 21/48 (44%) | 11/48 (23%) | 2/48 (4%) | 6/48 (13%) | 8/48 (17%) |
| Gender | Female | 23/48 (48%) | 10/21 (48%) | 3/11 (27%) | 1/2 (50%) | 4/6 (67%) | 5/8 (63%) |
| Age |  |  |  |  |  |  |  |
|  | Mean +/- SD | 9.8 +/- 8.8 | 8.6 +/- 4.3 | 7.1 +/- 6.1 | 9.0 +/- 11.4 | 21.2 +/- 17.2 | 8.4 +/- 7.7 |
|  | Median | 8.8 | 9.0 | 4.0 | 9.0 | 15.5 | 6.5 |
|  | Min | 0.0 | 2.0 | 0.0 | 0.9 | 3.0 | 0.0 |
|  | Max | 42.0 | 15.6 | 18.0 | 17.0 | 42.0 | 21.0 |
| Identified genetic pathogenic variation | |  | NA | 11/11 (100%) | 2/2 (100%) | 6/6 (100%) | 8/8 (100%) |
